# Stability of axis and patient satisfaction after toric implantable collamer lens implantation for myopic astigmatism

**DOI:** 10.12669/pjms.296.3986

**Published:** 2013

**Authors:** Tai-xiang Liu, Xin Luo

**Affiliations:** 1 Tai-xiang Liu, Zhongshan Ophthalmic centre of Sun Yat-Sen University, Guangzhou 510060, China. Department of Ophthalmology, Affiliated Hospital of Zunyi Medical College, Zunyi 563003, China.; 2 Xin Luo, Department of Ophthalmology, Affiliated Hospital of Zunyi Medical College, Zunyi 563003, China.

**Keywords:** Myopic astigmatism, Phakic eyes, Toric implantable collamer lens, Visual functions

## Abstract

***Objectives: ***To assess the stability of axis and patient satisfaction after toric visian implantable collamer lens (TICL) implantation for moderate to high myopic astigmatism.

***Methods: ***Total 33 eyes of 21 consecutive patients who underwent implantation of TICL for the correction of moderate to high myopic astigmatism were recorded and a minimum follow-up of six months was performed. The deviation of axis of TICL was detected from one week to six months postoperatively. The evaluation of the visual functions was done by the same clinician at six months after surgery.

***Results: ***The mean refractive cylinder decreased from -2.48±0.91 diopters (D) preoperatively to -0.54±0.25D and -0.50±0.19D at one week and six months after surgery respectively. Mean changes in astigmatism from one week to six months after surgery was 0.03±0.17D. The mean deviation of axis of TICL from one week to six months postoperatively was 2.48±1.25°(range,1°~ 6°) and no TICL required secondary repositioning. 14.3% patients felt difficult about the middle distant visual function (Reading Computer Screen). Evaluations of other visual functions were positive or very positive.

***Conclusions*** **: **Six months after implantation of the TICL, it showed slightly axis rotation and high satisfaction about the visual functions.

## INTRODUCTION

Refractive error is the very common eye disease around the world. The refractive surgery has become widely accepted as an effective method to correct the visual acuity. At present, refractive laser treatment such as laser in situ keratomileusis (LASIK) is still the main selection. However, both LASIK and photorefractive keratectomy (PRK) could induce astigmatism.^1^ Moreover a large amount of laser ablation may lead to the deterioration of optical performance of the cornea.^2^^-^^4^ It has already been demonstrated that the Visian Implantable Collamer Lens (ICL, STAAR Surgical, Nidau, Switzerland) implantation is better than LASIK in all measures of safety, efficacy, predictability, and stability.^5^^-^^8^ The ICL is a posterior chamber phakic intraocular lens (PC PIOL) made of collamer (collagen and a polyhydroxyethyl methacrylate-based copolymer), a flexible material. Recently the toric visian implantable collamer lens (TICL) has been demonstrated to be effective for the correction of high myopic astigmatism^9^^-^^11^ and that contrast sensitivity function was significantly improved.^12^ However, the rotational stability of TICL is still one of the main problems. Axis rotation after surgery may result in the deterioration of visual performance and consequently, patient dissatisfaction.

The aim of this study was to assess the clinical outcomes, including the stability of axis and patient satisfaction of the daily visual function after implantation of the TICL.

## METHODS

Thirty-three eyes (of 6 men and of 15 women) of 21 consecutive patients who underwent TICL implantation at Affiliated Hospital of Zunyi Medical College (Zunyi, China) from 2010 to 2011 with a minimal follow-up for 6 months after surgery were included in this study. The patient age at the time of surgery was 29.6 (7.3) (range, 22 to 41) years. The preoperative manifest spherical equivalent was -11.55 (3.52) (range,-5.75 to -16.25) D; The preoperative manifest refractive cylinder was -2.48 (0.91) (range, -1.25 to -4.5) D. Patients with anterior chamber depth of 2.8 mm or more, endothelial cell density of 2000 cells/mm2 or more, no history of ocular surgery, progressive corneal degeneration, cataract, glaucoma, or uveitis were included in our study.

TICL power was calculated using the software provided by the manufacturer (STAAR Surgical).^13^ The size of the TICL also was chosen by the manufacturer on the basis of the horizontal white-to-white and anterior chamber depth.

The patients underwent two peripheral iridectomies with a neodymium: yttrium–aluminum– garnet laser (Nd:YAG) one week before surgery. On the day of surgery, the patients were dilated. The 0°~180° axis and the desired axis of TICL were marked before surgery using a slit lamp. After topical anesthesia, a V4 TICL was inserted into the anterior chamber and the axis of TICL was placed at the marked position. After surgery, steroidal and antibiotic medications were used topically four times daily for two weeks. The axis of the TICL was measured at the slitlamp following mydriasis at one week and six month after surgery. The axis of the slit-beam was aligned along the TICL axis and the angle of rotation recorded. Examination at one week postoperatively was regarded as the baseline of axis alignment. The rotational degrees of TICL were the absolute difference value of angle of TICL from one week to six months after surgery. Questionnaire of visual function^14^ was administered to assess patient satisfaction about the outcome six months after implantation of TICL (Table-I).

**Table-I:** Patients subjective feeling of daily visual functions six months after implantation of TICL.

*Visual functions*	*Responses % (No. Patients)*	*Mean response ±SD*	*Very positive % (No. Patients)*	*Positive% (No. Patients)*	*Negative % (No. Patients)*	*Very negative % (No. Patients)*
Reading in daylight	85.7 (18)	8.4±0.8	83.3(15)	16.7(3)	0(0)	0(0)
Reading in artificial light	85.7 (18)	7.8±1.0	50(9)	50(9)	0(0)	0(0)
Watching TV	100 (21)	7.9±1.0	57.1(12)	42.9(9)	0(0)	0(0)
Watching movie at cinema	71.4 (15)	8.0±1.0	60(9)	40(6)	0(0)	0(0)
Driving in daylight	100 (21)	7.9±1.0	57.1(12)	42.9(9)	0(0)	0(0)
Driving at night	85.7 (18)	7.9±1.4	66.7(12)	33.3(6)	0(0)	0(0)
Reading computer screen	100 (21)	7.8±1.3	57.1(12)	38.1(6)	14.3(3)	0(0)
Playing sports	100 (21)	7.9±1.0	57.1(12)	42.9(9)	0(0)	0(0)
Swimming	100 (21)	7.9±1.0	57.1(12)	42.9(9)	0(0)	0(0)
Shaving	57.1 (12)	7.8±1.1	60(9)	40(6)	0(0)	0(0)
Applying makeup	85.7 (18)	7.8±1.0	50(9)	50(9)	0(0)	0(0)
Shopping	100 (21)	8.2±0.9	71.4(15)	28.6(6)	0(0)	0(0)
Seeing uncorrected upon awakening	100 (21)	7.9±0.9	57.1(12)	42.9(9)	0(0)	0(0)

Statistical analyses were performed using SPSS for Windows (SPSS, Inc, Chicago, IL, USA. version 17.0). The results are expressed as mean±SD and a value of *P<*0.05 was considered statistically significant.

## RESULTS

***1: The residual refractive error: ***The mean residual astigmatism was -0.54±0.25D (range, 0 to -1.0D) and -0.50±0.19D (range, 0 to -0.75D) at one week and six months respectively (Table-II). The mean change of astigmatism was 0.03±0.17D from one week to six months after surgery. The refractive cylinder decreased by 78.2% and 79.8% at one week and six months postoperatively. 

The mean spherical equivalent was -0.71±0.39D and -0.68±0.36D at one week and six months respectively. One week and six months after surgery, 63.6% and 63.6% of eyes and 81.8% and 90.9% of eyes were within ±0.5D and within ±1.0D.

**Table-II:** The cylinder after toric implantable collamer lens implantation

*Cylinder (D)*	*Preoperative (n)(%)*	*1 Week post- (n)(%)*	*6 Months post- (n)(%)*
≤-0.25	0 (0)	18(54.6)	18(54.6)
≤-0.5	0 (0)	27(81.8)	27(81.8)
≤-0.75	0 (0)	30(90.9)	33(100)
≤-1.0	0 (0)	33(100)	33(100)
≤-1.5	3 (9.1)	33(100)	33(100)
≤-2.0	15 (45.5)	33(100)	33(100)
≤-2.5	24 (72.7)	33(100)	33(100)
≤-3.0	24 (72.7)	33(100)	33(100)
≤-3.5	30 (90.9)	33(100)	33(100)
≤-4.0	30 (90.9)	33(100)	33(100)
≤-4.5	33 (100)	33(100)	33(100)

**Fig.1:**
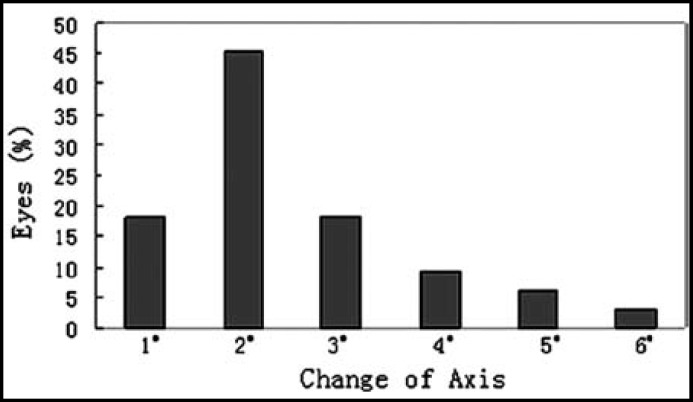
The** c**hange of axis of TICL from one week to six months after surgery

***2: The change of axis of TICL: ***The mean TICL rotation was 2.48±1.25° from one week to six months after surgery (Fig.1). The clockwise (12 eyes) and counterclockwise (21 eyes) deviation were 2.76±1.51 and 2.0±0.74°respectively. No TICL required secondary repositioning.

**Fig.2:**
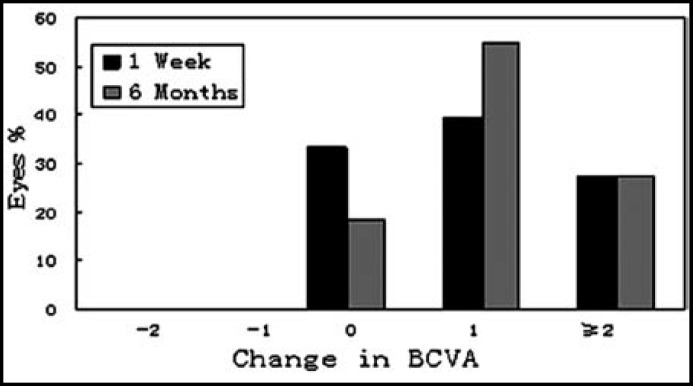
The change in BCVA one week to six months after TICL implantation

***3: Visual acuity: ***The best corrected visual acuity (BCVA) of all eyes was 0.6 or more after surgery. The safety index was 1.20±0.22, 1.25±0.23 at one week and six months after surgery, respectively. No eye lost 1 line or more; twenty-two eyes (66.7%) and twenty-seven eyes (81.8%) gained 1 line or more at one week and six months after TICL implantation respectively (Fig.2).

**Fig.3:**
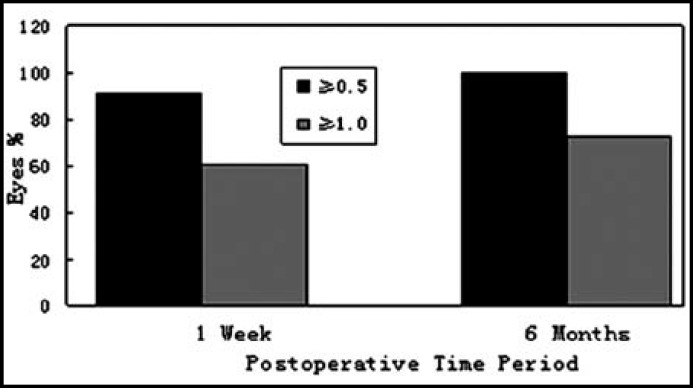
The change in UCVA one week to six months after TICL implantation

At one week and six months after TICL implantation the uncorrected visual acuity (UCVA) was 0.4 and 0.5 or more respectively (Fig.3). The efficacy index was1.06±0.32 and 1.09±0.20 at one week and six months after surgery. At one week and six months after surgery, the UCVA of 69.7% eyes and 81.8% eyes was equal or superior to the preoperative best corrected visual acuity respectively. 

***4: Visual function after surgery: ***At six months after TICL, patients were invited to mark the questionnaire (Table-I) and some questions had no response. Overall, three patients (>36 years old) expressed negative about Reading Computer Screen (middle distant visual acuity) and the other responses were positive or very positive. Based on the age, only the mean response of the younger age group (≤35 years old, n=12) about Reading Computer Screen was higher than that of the older group (≥36 years old, n=9) (monovariate t test, P=0.04).

## DISCUSSION

In our study, the data showed that TICL implantation were good in all measures of stability of the axis of TICL and patient satisfaction about the visual function six months after surgery.

We observed the residual astigmatism for the TICL implantation. Our results indicated that manifest astigmatism was significantly reduced. The cylinder decreased by 79.8% six months post-operation, which was similar to the previous studies.^10^^,^^15^ No eye decreased in UCVA from one week to six months after treatment. This meant that the recovery of visual acuity was quick and this procedure had good efficacy and predictability.

The rotation of the axis is the main problem for the TICL, which will result in axis shift and reduced efficacy of astigmatic correction.^16^ To assess the stability of axis after TICL implantation, the axis of TICL at one week after surgery was considered the baseline value. The rotational degrees of TICL were the absolute difference value of angle of TICL from at one week to six months after surgery. The result of our study at six months showed that the mean rotation of TICL was 2.48±1.25°. This was supported by previous studies.^10^^,^^15^^,^^17^ If the TICL rotates away from its intended axis, astigmatic change occurs. The power of the new cylinder depends on the angle of rotation and on the power of the toric IOL.^18^ In our study; the mean change of astigmatism was 0.03±0.17D from one week to six months after surgery. It indicated that the axis of TICL had good stability after implantation.

Patient satisfaction about the visual functions is one of the focuses of refractive surgery. Our questionnaire showed that most patients felt positive or very positive about the visual functions after TICL implantation. The procedure could satisfy most patients’ daily visual functional activities. However, there still were some patients in the elderly group, who felt difficulty about the middle distant visual function (Reading Computer Screen). At present, we have no clear explanations for this phenomenon. Unfortunately, there is still no proper method to evaluate and improve the middle distant visual function. Because of nature of their jobs and studies, patients who are appropriate group for the TICL implantation use the middle distance vision very frequently. Maybe it is time to pay attention to the middle distance visual acuity after refractive surgery.

In our six months follow-up after TICL implantation, the axis of TICL still had slight rotation, and no patient required secondary repositioning. Maybe the slight rotation of TICL will not significantly affect the visual performance and patient satisfaction. However, future long-term and larger patient samples investigation about the stability and patients’ satisfaction should be performed.
